# Direct and indirect effects of plant diversity and phenoxy herbicide application on the development and reproduction of a polyphagous herbivore

**DOI:** 10.1038/s41598-020-64252-5

**Published:** 2020-04-29

**Authors:** Yeisson Gutiérrez, David Ott, Christoph Scherber

**Affiliations:** Institute of Landscape Ecology, University of Münster, 48149 Münster, Germany

**Keywords:** Biodiversity, Ecophysiology, Grassland ecology

## Abstract

Widespread application of synthetic pesticides and loss of plant diversity are regarded as significant drivers of current global change. The effects of such phenomena on insect performance have been extensively studied separately, yet the interactions of these two drivers have been poorly explored. Here, we subjected the polyphagous grasshopper *Pseudochorthippus parallelus* (Zetterstedt, 1821) to a full-lifecycle field experiment with 50 cages containing experimental plant communities differing in grass species richness (2 vs. 8 grass species), half of them treated with a phenoxy herbicide commonly employed to control broadleaf plants in grasslands. We measured plant elemental content as a proxy for plant physiology, and a wide range of insect traits in both female and male grasshoppers. In females, grass diversity increased herbivory, insect nitrogen content and egg load, while herbicide reduced herbivory but increased the number of offspring, likely mediated by altered plant community composition. In males, grass diversity also increased herbivory, had positive effects on fat body, muscle volume and lifespan, and negative effects on body mass. Herbicide negatively affected herbivory in both females and males. Overall, plant diversity and herbicides may shift resource allocation in generalist terrestrial insect herbivores, indicating complex and unexpected effects of human-induced environmental change.

## Introduction

Agricultural management techniques may impact living organisms in different ways. Grasslands have historically been subjected to activities that strongly affect plant community composition, such as cutting, grazing and burning^1,2^. In a similar manner, herbicide application (e.g. triazine and phenoxy classes) has been an effective and widely used method to maintain grass dominance in this biome^3–5^.

Changes in plant communities bear the potential to directly or indirectly affect all trophic levels and ecosystem processes^6,7^. Consequently, the ecological effects of plant community simplification (i.e. species richness loss) have been explored in great detail at the population and community scale^7–11^. Nevertheless, mechanistic studies on the effects of plant community simplification have yielded inconsistent results: while some studies have demonstrated positive effects of increased plant diversity on insect reproductive output^12–14^, other studies with a similar approach have shown no effects at the individual level^15–17^. Therefore, a thorough understanding of the relationship between plant diversity and herbivore performance is the first knowledge gap that we address in the present study.

On the other hand, toxicological effects of herbicides have been fairly well studied from a mechanistic point of view, as herbicides can exert unintended effects on non-target organisms (e.g. insects and microorganisms)^18^. Yet, the effects on ecological interactions under field conditions have been almost neglected so far (but see^19–21^). In this study, we focus on a particular herbicide class, namely phenoxy herbicides, which target mainly dicotyledonous plants^22^, a characteristic that makes them suitable for grassland management^2,23,24^. This herbicide class is among the most frequently used herbicides in conventional grassland management^24–26^. Direct contact (i.e. topical application) of several products belonging to this herbicide class cause detrimental effects in a variety of animals, including lady beetles^27–29^, bees^30,31^, moths^32^, earthworms^33^ and even vertebrates^34,35^. However, phenoxy herbicides are usually applied to grassland early in the vegetation period or after (re-)sowing^36,37^, hence direct contact to insects might be rare under field conditions of this application scenario. Studies on indirect herbicide effects on insects even showed enhanced reproductive output in insects fed with plants treated with phenoxy herbicides^38,39^. Such a phenomenon has been suggested to be related to induced physiological changes in the host plant, mainly associated with alteration of the content of total nitrogen, free amino acids and protein^40–42^.

Here, we used a field experiment to subject a generalist grasshopper to experimental manipulation of plant diversity crossed with herbicide application. To separate aboveground plant diversity effects from indirect effects of root competition, we manipulated plant diversity using potted plants in an experimental grid array to resemble a plant community simplification event. In addition, a subset of the plants was treated with a phenoxy herbicide (regarded as non-toxic to grasses) prior to the introduction of the herbivores in an attempt to elicit plant physiological changes. To our knowledge, no study to date has explored the effects of plant community simplification and herbicide treatment in combination on the development and reproduction of a herbivorous insect.

The grasshopper *Pseudochorthippus parallelus* (Zetterstedt, 1821) (Orthoptera: Acrididae: Gomphocerinae) was used as a focal species, as it is common in Central European grasslands^43,44^. The species has been used as a model organism in several other studies on plant-insect interactions in the context of grassland species diversity^15–17^ and extensive work has been done on the dietary preferences of this insect^45–47^. Furthermore, the species is ecologically relevant as grasshoppers are the most dominant invertebrate herbivore in grassland biomes and are important for ecosystem functioning, such as nutrient and energy cycling^48–51^.

We hypothesize that an enriched plant (grass) community (i.e. higher species richness) would benefit the insects by offering a wider range of resources to achieve nutritional optima and maximize fitness. Additionally, we expected the herbicide to induce physiological changes in the plants (i.e., altered quality), which in turn would be reflected in differences in insect traits. We tested these hypotheses by measuring plant physiological traits, and sex-specific developmental, morphological and physiological traits of the grasshopper *P. parallelus* feeding in experimental plant communities.

## Results

### Model specification

From all the initially considered insect traits, development time and survival were discarded from the models as insects exhibited little variation in such traits and these did not improve model fit. Although both insect nitrogen and carbon were measured, we included only insect nitrogen in the final models because carbon or C/N ratio did not add explanatory power to the models. Non-significant paths were removed and alternative paths were added in models using the conceptual model as a starter. The initially proposed latent variable “insect body condition” was not supported by any of the alternative models specified; therefore, we considered insect weight and volume as separate endogenous variables.

**Figure 1:**
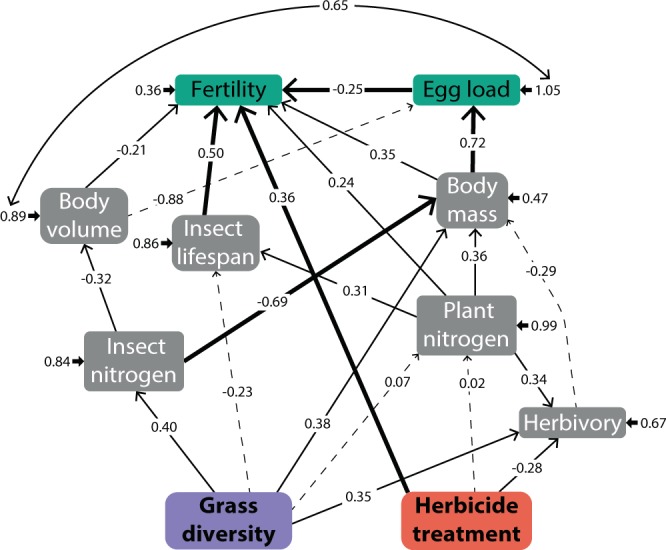
Structural Equation Model (SEM) showing direct effects of the experimental treatments, plant community diversity and herbicide treatment, on the performance and reproduction of female *P. parallelus*. Each arrow is accompanied by standardized path coefficients. Arrows format indicates statistical significance, bold for P < 0.001, thin for P < 0.1 and dashed for non-significant relationships. Short arrows are error terms. All P-values for path coefficients are available in Supplementary Table S2.

**Figure 2:**
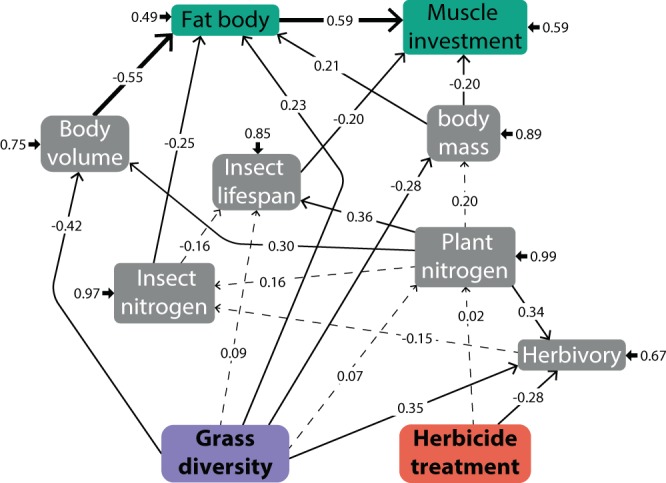
Structural Equation Model (SEM) showing direct effects of the experimental treatments, plant community diversity and herbicide treatment, on the performance of male *P. parallelus*. Each arrow is accompanied by standardized path coefficients. Arrows format indicates statistical significance, bold for P < 0.001, thin for P < 0.1 and dashed for non-significant relationships. Short arrows are error terms. All P-values for path coefficients are available in Supplementary Table S3.

Herbivory was measured as a single response in every experimental cage, but was included in each sex-specific model (with the same values) as it appeared to be an important component in the system dynamics (according to model fit parameters). Overall, herbivory was positively influenced by plant community diversity and plant nitrogen content, and negatively influenced by herbicide application (Figs. 1 and 2). A more detailed analysis of plant species responses to herbicide application evidenced that plant biomass was affected in a species-specific manner (Supplementary Fig. S3). The biomass produced by two plant species was positively affected by herbicide application, *Dactylis glometara* (Poaceae) and *Trifolium repens* (Fabaceae); from which, only *T. repens* was present in all experimental cages. Although changes in biomass in this particular species (i.e. *T. repens*) may have directed the positive effects of herbicide application, there was no significant difference in the consumption of herbicide-treated and untreated *T. repens*.

### Female Structural Equation Model (SEM)

**Figure 3:**
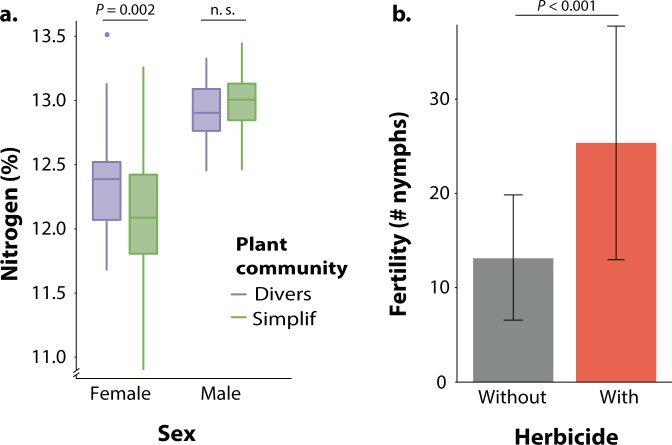
(**a**) *Pseudochorthippus parallelus* nitrogen content when fed with diversified and simplified experimental plant communities. (**b**) Fertility of female *P. parallelus* when fed with plants treated with a phenoxy herbicide. Variation expressed as confidence intervals.

The structural equation model (Fig. 1) had X^2^ = 8.29 (d.f. = 10.33, *P* = 0.630, non-significant *P* values indicate good fit). The full results of the model and total effects (combining direct and indirect effects) are presented in Supplementary Table S2. Experimental treatments significantly affected female *P. parallelus* development and reproduction. On the one hand, plant community diversity positively influenced insect nitrogen content and weight (Fig. 3a, Supplementary Table S2), yet the total effect of plant community diversity on female weight was almost negligible (Supplementary Table S2). On the other hand, herbicide application had a positive direct effect on female *P. parallelus* fertility, which was apparently not related to an indirect effect on any other insect trait (Fig. 1, Supplementary Table S2). Plant nitrogen content was not significantly affected by any of the experimental treatments. Yet, this variable positively influenced many female *P. parallelus* developmental and reproductive traits (Fig. 1, Supplementary Table S2).

All considered, fertility was strongly influenced by several traits in addition to the experimental factor herbicide application (Figs. 1 and 3b). All these traits, including plant nitrogen content and insect lifespan, as well as insect weight, had a positive influence on fertility; only initial egg load had a negative influence (Supplementary Table S2). Notably, lifespan, which was positively influenced by nitrogen content only, had the strongest effect on *P. parallelus* fertility (Supplementary Table S2).

### Male Structural Equation Model (SEM)

**Figure 4:**
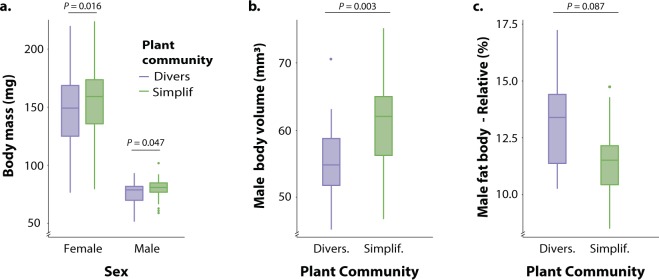
(**a**) Body mass of *Pseudochorthippus parallelus* when fed with diversified and simplified experimental plant communities. (**b**) Male *P. parallelus* body volume when fed with diversified and simplified experimental plant communities. (**c**) Male *P. parallelus* fat body volume (relative to body size) when fed with diversified and simplified experimental plant communities.

The structural equation model (Fig. 2) had X^2^ = 5.32 (d.f.= 11.64, *P* = 0.936, non-significant *P* values indicate a good fit). The full results of the model and total effects (combining direct and indirect effects) are presented in Supplementary Table S3. Plant community diversity negatively affected insect total weight (Fig. 4a) and volume (Fig. 4b), but had a positive influence on relative fat body volume (significant at *P* = 0.086, Fig. 4c, Supplementary Table S3) and muscle investment (in an indirect manner, Fig. S4, Supplementary Table S3). In contrast, herbicide application did not affect any relevant trait of male *P. parallelus* neither directly nor indirectly. Plant nitrogen, similar to females (above), was not affected by any of the experimental treatments. Nevertheless, it positively influenced lifespan and insect volume (Fig. 2, Supplementary Table S3).

Relative investment in muscle tissue was influenced negatively by body mass and lifespan. On the other hand, relative fat body was influenced positively by body mass, yet negatively by body volume. While both proxies of male *P. parallelus* performance (i.e. relative fat body volume and investment in muscles) had a positive relationship, the direction of such a relationship cannot be clearly established with the current dataset (double-headed arrow) (Fig. 2. Supplementary Table S3).

## Discussion

Our study integrated a wide range of insect traits to shed light on the mechanistic understanding of insect responses to two interacting stressors commonly occurring in managed grasslands. We found that the polyphagous herbivore *P. parallelus* was affected by the experimental treatments in direct and indirect ways in a sex-specific manner; which is in line with previous studies that have suggested sex-specific resource requirements and investment due to divergent life-history strategies in males vs. females^52–54^. Plant community diversity affected female C/N ratio only, yet the effects on males were more complex. Male *P. parallelus* had a smaller body size and increased capacity to store energy and invest in muscle tissue when having access to diversified plant communities. Conversely, herbicide treatment did not affect male traits, yet had a noticeable positive effect on female fertility.

Moreover, smaller body size in male *P. paralleus* fed with diversified plant communities could have been perceived as an indication of malnutrition^55^. However, smaller males invested more in fat storage and muscle tissue when fed with diversified plant communities, thus indicating a different energy allocation strategy^56^. While smaller female size has been commonly associated with reduced reproductive output, in males, the relationship between size and fecundity is less clear^57,58^. For instance, in a study carried out by Pitnick^59^, small *Drosophila* males had higher fecundity than bigger males. Yet, In several orthopteran species, females are known to choose males based on their size^60–62^. Additionally, the size of male grasshoppers has been shown to be positively correlated with important traits of singing behavior^63^ and offspring size^64^. Most of the aforementioned arguments seem to indicate that a smaller size may be detrimental to the performance of male *P. paralleus*.

Regarding female *P. paralleus* body condition, weight was unaffected by plant community diversity but females had increased nitrogen content at a higher plant diversity. Surprisingly, in our study, nitrogen content was negatively correlated with female body size. Although nitrogen has been found to be important for grasshopper reproduction^65^ there seems to be a non-linear relationship between available nitrogen and reproduction^66^. Further, it is possible that other limiting elements (e.g. phosphorus) may play an important role in the allocation of nutritional resources for egg production;^67,68^ this will need to be elucidated in further studies.

In contrast to the strong effects on nitrogen content, plant diversity did not significantly affect fertility in female grasshoppers. In studies carried out by Pfisterer *et al*.^14^ and Unsicker *et al*.^13^, the positive effect of plant diversity on female reproductive output was apparent when grasshoppers had access to more than 30 plant species from diverse families and functional groups. Such benefits could presumably have been caused by a dietary shift in the grasshoppers (i.e. by including more legume and herb species in their diets)^14^. In our study, we employed a gradient in grass species richness (resembling intensively farmed grassland^69^), indicating that strong biodiversity effects on female reproductive output may not be found in intensively managed grasslands.

Regarding the positive effect of herbicide treatment on female fertility, our results do not allow us to establish a mechanism for such an effect. Initially, we had hypothesized that plant physiology would be changed due to the stress imposed by the herbicide, and this would increase forage quality as suggested by previous studies^40,41,70^. However, additional analyses showed that plant nitrogen content (a proxy for potential physiological changes in response to stress (reviewed by Jones^71^) was not affected by herbicide application. For instance, the most widely used phenoxy herbicide, 2,4-D, has proven to increase nitrogen content in wild garlic, *Allium vineale* L.^70^, increase protein content in wheat, *Triticum aestivum* L.^40^ and changed the amino acids profile of potato tubers^41^.

When plants receive an application of a phenoxy herbicide, the herbicide molecules are transported mainly to young leaves, leaf veins, roots, and nodules, where the metabolism of this herbicide occurs^72,73^. Previous studies have shown that after three weeks following herbicide application, about 20-30% of the initial concentration sprayed is still present in grass tissues^74,75^, and about 25% is still traceable in *Trifolium repens*^76^. However, the grasshoppers used in this study were in contact with the treated plants long after the herbicide application, and they were actively feeding on the plants while these were still in full development. Therefore, we consider it unlikely that the insects ingested a significant amount of these (potentially toxic) compounds. Furthermore, even when phenoxy herbicides would have been ingested, these compounds would not accumulate in animal tissue, but rather be rapidly excreted (reviewed in^77^).

Based on our findings, we would rather hypothesize that plant physiology was altered after herbicide treatment, and this phenomenon turned out to be beneficial for the female *P. parallelus*. However, this linkage, and in particular plant physiological changes, should be studied more thoroughly in the future.

Overall, studies on the effects of herbicides (in general) on invertebrates are relatively scarce^18^ and most reports on toxicological effects on phenoxy herbicides were the results of assays of topical (i.e. applied directly to a part of the body) and ingestion assays in vertebrate species^78,79^. Nevertheless, a handful of studies have shown that overall, phenoxy herbicides cause negative (commonly lethal) effects on insects after topical application^27–29,31,32,42,80^.

However, early field studies suggested that crops treated with phenoxy herbicides experienced an increase of herbivorous insect populations^81,82^. Yet, the authors of such studies attributed this phenomenon to a possible negative impact of the herbicide on natural predators. Later, it was shown that herbicide-treated (2,4-D) broad beans plants led to an increased reproduction capacity of the pea aphid^38^, implying that such aphids were benefitted through an indirect effect of the herbicide on host-plant quality. Such an effect may be related to the increased content of amino acids and proteins reported in other studies^40,41,70^.

Moreover, plant consumption (i.e. herbivory) was greater with increased grass diversity, yet it was negatively influenced by herbicide treatment. Nevertheless, the legume *T. repens* had an increased biomass when treated with the phenoxy herbicide. This phenomenon likely affected grasshopper feeding, yet, further experiments would be needed to test this mechanism in greater detail.

In natural plant communities, herbivory has been found to either decrease^83,84^ or increase^85^ with overall plant species richness; however, these findings cannot directly compared with our study, as community-level invertebrate herbivory in real grasslands is usually caused by a mixture of specialist and generalist herbivores. In our study, each grass species grew physically isolated from other species in pots, excluding indirect belowground effects or aboveground plant competition for light. Hence, differences in herbivory were likely not indirectly caused by differences in plant productivity as in other biodiversity-ecosystem functioning studies^86^. Additionally, other studies have shown that other plant community traits (e.g. functional diversity) would be more influential on herbivory than mere plant diversity^85^. It is worth mentioning that although our herbivory estimation did not consider possible non-additive (i.e. synergistic or antagonistic) effects of herbicide and herbivory effects on plant physiology, these two plant stressors might modulate the insect-plant interaction by the stress induced by herbicide application (e.g. overcompensation)^87,88^.

### Conclusion

Our study showed that herbicides had a strong effect on relevant insect traits (e.g. size and fertility). Additionally, we found that plant species richness affected the performance of insects in a sex-specific manner, challenging previous studies that had asserted that plant diversity would not directly benefit individual performance^15,16^.

Although pesticides can exert significant and (in some cases) long-lasting consequences in natural and managed ecosystems, they have been highly disregarded as global change drivers. Only recent studies have started to highlight their importance in shaping ecosystem processes and functions^89,90^. Our results are in accordance with previous studies that have shown beneficial effects of phenoxy herbicides on herbivore performance. However, the mechanisms for such effects remain elusive, and the potential direct and indirect effects of such herbicides on other organisms (specialist herbivores, decomposers) and ecological relationships warrant further study.

## Materials and Methods

### Rearing of study organisms

Approximately 500 adults *P. parallelus* were collected in an Arrhenatheretum meadow in Jena (Germany) in early August 2016. The insects were kept under laboratory conditions and a mixture of grasses was offered *ad libitum* and replaced bi-weekly. Small plastic containers (d = 10 cm) filled with wet sand and soil (1:1) were offered as oviposition substrate. The egg-carrying containers were kept in a climatic cabinet (ET 650-8, Aqualytic, Germany) at 4 °C from September 2016 to May 2017. Subsequently, the temperature was raised to 26 °C in gradual increases and the grasshoppers hatched after 11-13 days. The hatchlings were kept under laboratory conditions (12:12 h, 20 ± 2 °C, 55 ± 5%RH) and fed with a mixture of field-collected grasses (mainly *Dactylis* sp.) until the second instar was reached (see below).

Seeds of 14 grass species (Poaceae) and *Trifolium repens* L. (Fabaceae) were purchased from a commercial supplier (Rieger-Hofmann GmbH, Germany). The selection of the grass species was based on known acceptance by the grasshopper *P. parallelus*^12,46^ and because these species were abundantly present in local grasslands. The experiment setup allowed for no physical interaction between roots of the plants, therefore we assume no effects of plant diversity treatment on herbivory or plant traits.

### Experimental design

Second instar *P. parallelus* were added to cages containing experimental plant communities of high vs. low grass species richness that were either herbicide-treated or not (N = 2 diversity levels x 2 herbicide treatments x 8 replicates = 32). Plant communities were drawn at random from a pool of 14 grass species and were either species-poor (two grass species) or species-rich (eight grass species) and always contained one pot of *T. repens* (Fabaceae) (substitutive design, i.e. constant density). Selection of grass species at random from the species pool circumvented plant identity as a potential confounding factor. Additionally, 16 cages were installed that contained no grasshoppers (used for control measurements on plants) (Supplementary Fig. S1). A phenoxy herbicide was applied at random to half of the experimental communities; this herbicide is selectively toxic to broadleaf plants^37,73^.

The simplified community and the usage of *T. repens* as representative of the legumes for this experiment were chosen based on results of Unsicker *et al*.^12^ who demonstrated that the grasshopper *P. parallelus* cannot successfully complete the development under monospecific feeding and a minimum taxonomic diversity of plants is required. A layout of the experiment setup (Supplementary Fig. S2) and the complete list of plant species is provided in the Supplementary Information (Supplementary Table S1).

### Experiment setup

**Figure 5:**
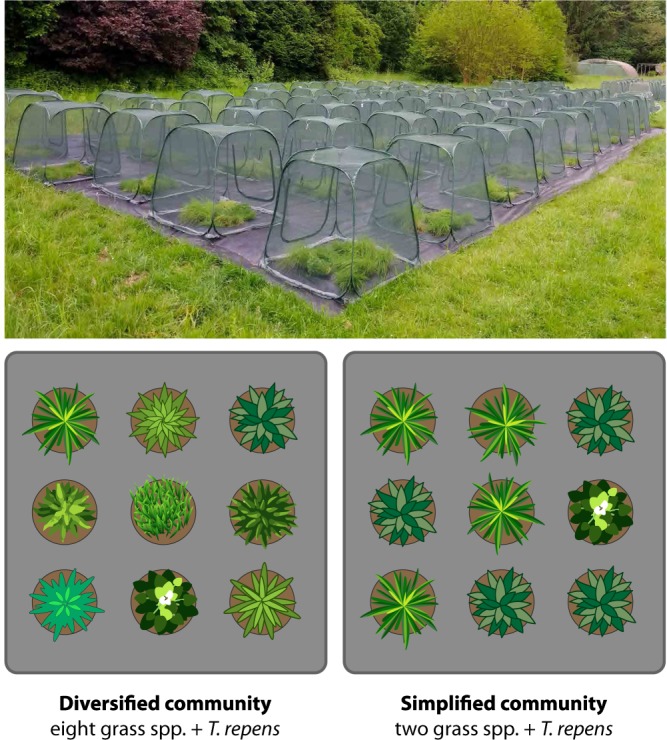
Experiment setup. Schematic representation of cages containing diversified (8 grass spp. + *T. repens*) and simplified (2 grass spp. + *T. repens*) experimental plant communities (a subset of such communities received herbicide and the remaining were left untreated).

Cages measuring 1 m^3^ with 1 mm^2^ mesh size (Nature Pop-upkas Anti-insect, Experty, The Netherlands) were installed in May 2017 in the Pharmaceutical Garden at the University of Münster, Germany (51°57′55“N 7°36′22“E). The area was lined with polypropylene geotextile (Hermann Meyer KG, Germany) to suppress weed growth and coerce the grasshoppers to oviposit inside the plant pots. Nine pots were installed in every cage, four pots of every grass species in the simplified community (2 species, 8 pots) and one pot per grass species in the diversified community (8 species, 8 pots). Additionally, one pot containing *T. repens* was added to every cage (Fig. 5).

Approximately 400 seeds of each species were sown in every 1.5 L plastic pot (d = 15.1 cm, h = 11.9 cm) using garden compost (TKS 1, Floragard, Germany) with the following chemical properties: pH 5.6, salt content 0.8 g/L, nitrogen 140 mg/L, phosphate 80 mg/L, potassium 190 mg/L, PG-Mix 18-10-20 0.8 kg/m^3^. Pots were partially burrowed to assure easy access to the grasshoppers (especially in the early nymph stages) and to avoid root damage because of warm-up^91^.

Five pots of every plant species (with and without herbicide application) were used as a control to estimate the effects of herbivory during the development of the grasshopper nymphs. These potted control plants were randomly positioned inside N = 12 cages to exclude herbivores and were subjected to the same environmental effects as the cages containing grasshoppers.

All plants were about two and a half months old when placed inside the cages. The experiment was initiated in late May 2017 when every cage was stocked with 14 second-instar *P. parallelus* (hatched in lab conditions, see above). All potted plants were replaced after one month with a second batch that had received the same treatment (replacements took place when most of the insects had reached adulthood). The first batch of plants was used for measurements of plant biomass (i.e., estimation of herbivory) and C/N ratio (used as a proxy for changes in plant physiology^71^). The second batch of pots was overwintered in the experimental setup outdoors after the natural death of the adult insects (ca. five months after onset). In early spring 2018, the potted plants were relocated to a greenhouse to monitor the hatching of eggs.

The selective herbicide used was a combination of 2,4-DB and MCPA (Clovermax, Nufarm, The Netherlands). The product was applied at the recommended field rate 7 L/ha using a calibrated garden sprayer (828-20, Gardena, Germany). Thus, 3.00 mg of 2,4-DB and 0.50 mg of MCPA active ingredient (nominal concentrations) were sprayed to every pot using tap water as solvent three weeks before the onset of the experiment (early May 2017, amounts calculated after the surface area of the pot). All pots were sprayed on the same day during the early morning to avoid excess evaporation; every pot received the aforementioned amount of Clovermax herbicide diluted in 40 mL tap water.

### Measurement of Development and fertility

All insects were weighed to the nearest 0.001 g (Kern Präzisionswaage 572-30, Kern, Germany) upon reaching adulthood and marked with acrylic paint to accurately calculate the age for subsequent analysis. A subset of grasshoppers was kept in the cages until natural death to determine lifespan and fertility (i.e., nymphs hatching after overwintering period relative to the number of females per cage, experimental procedure explained above).

### Morphological analysis and C/N ratio

Eight-days-old adult males and ten-days-old adult females were collected from every cage for C/N analysis (N = 112) and internal morphology (N = 63). This standardized age for insect collection was selected using the maturation time of *Omocestus viridulus* (L. 1758) (another species from the subfamily Gomphocerinae) as a proxy for full body development just before the first oviposition event^92^. Besides, age standardization allowed for confident comparison of C/N composition, as it is known that it may vary with age and physiological status^93,94^.

#### Micro-computed tomography

Every specimen was scanned using Micro Computed Tomography (µ-CT). Equipment and methods for specimen preparation are described in the detail by Gutiérrez *et al*.^95^. In brief, specimens were fixed in FAE solution (Formaldehyde, acetic acid and ethanol), subsequently stained in 1% Iodine solution for 24 h and subjected to critical point drying. Scans were reconstructed using the ASTRA toolbox^96^, and slices were semi-automatically segmented using the software Segd3D^97^ and Biomedisa^98^.

Tissues were measured as volumes using a fixed voxel size of 10.25 µm for all specimens. The semi-automatic segmentation was contrasted against manually segmented slices and the lowest accuracy was 97.6%. The volume of the measured organs and muscles was relativized according to each individual volume.

**Figure 6:**
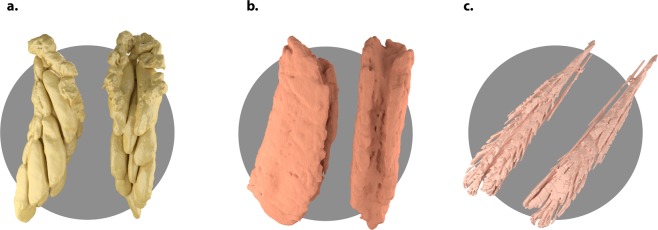
Internal organs of *Pseudochorthippus parallelus* measured using x-ray computed tomography. (**a**) The initial egg load was measured as the volume of egg carrying ovaries. (**b**) In males, we measured fat body volume (this tissue was almost inexistent in females at this developmental stage). (**c**) Femora muscle volume (extensor and flexor together) was measured as a proxy for investment in muscle tissue in both sexes.

We used micro-CT methodology to measure the total volume of each specimen and the following traits; in females, initial egg load was measured as the volume of egg carrying ovaries (Fig. 6a); in males, we measured fat body volume (Fig. 6b, this tissue was almost inexistent in females at this developmental stage) and femora muscles (Fig. 6c, extensor and flexor together) as a proxy for investment in muscle tissue in both sexes^99,100^.

#### Carbon and Nitrogen composition of insects and plants

Insects of the desired age (eight-days-old adult males and ten-days-old adult females) where freeze-killed and stored at -20 °C. At a later phase, specimens were allowed to defrost for 10 min, the gut of each specimen was removed to avoid contamination due to residuals of plant tissue, and all carcasses were dried for 48 h at 60 °C. Dry insects were individually ground to fine powder with a ball mill (MM400, Retsch, Germany) and total carbon and nitrogen were measured by using an elemental analyser (EA 3000, EuroVector, Italy). Carbon (C) and Nitrogen (N) percentage and the C/N ratio were used as response variables.

Plant matter was dried for 48 h at 60 °C, grounded using a cyclone mill (Cyclotec 1093, Foss A/S, Sweden) and analysed for nitrogen concentration by near-infrared spectroscopy (Spectra Star 2400, Unity Scientific, USA). The concentration was derived after calibration models covering a spectra range from 1250 to 2350 nm^101^ and the accuracy of the measurements was confirmed with a subset of samples measured with an elemental analyser (EA 3000, EuroVector, Italy).

### Estimation of herbivore consumption rates

The first batch of potted plants, exposed to grasshoppers for over a month, was collected for estimation of plant consumption under the experimental treatments (details above). All plants from every pot were clipped at a height of 2-3 cm above ground and dried for 48 h at 60 °C before weighing.

Consumption of aboveground plant biomass was calculated for every plant species independently as C - H ∀ H ≤ C, where C is the average dry biomass of the control potted-plants and H is the dry biomass of the plants that were inside cages with the grasshoppers *P. parallelus*. Total plant consumption per cage was calculated by adding the values of all 9 pots.

### Data analysis

**Figure 7:**
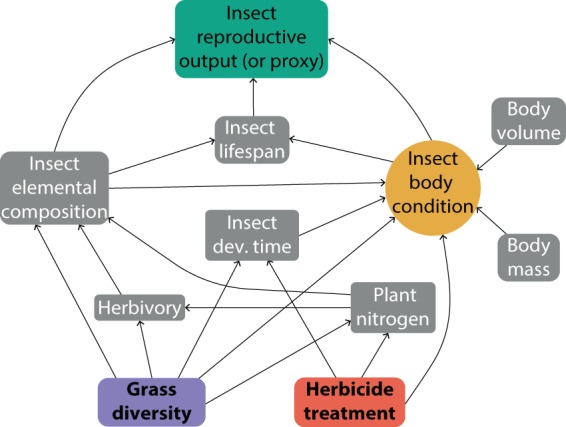
Conceptual Structural Equation Model (SEM) used both female and male *Pseudochorthippus parallelus*.

The analysis was performed using Structural Equation Modelling (SEM) in R^102^. Data were integrated into two datasets, one for each sex, due to the difference in measured traits, yet a similar conceptual model (i.e. structural equation meta-model) was used in both cases (Fig. 7). Average responses were calculated for each experimental cage and all variables were scaled to a numeric range of {0:10}^103^.

Models were fitted using the lavaan library^104^ with maximum likelihood estimation with robust standard errors and a mean- and variance adjusted test statistic (MLMVS) to account for heteroscedasticity in the data. We tested for direct and indirect effects of the main experimental treatments on the performance and reproduction of the grasshopper *P. parallelus*. The exogenous variables were the experimental factors (herbicide application and plant community diversity), which were converted to binary and assumed to be fixed (without associated error terms). Plant nitrogen content and herbivory were used as moderator variables.

All insect traits measured during the experiment were integrated into the model as response (endogenous) variables. Additionally, we hypothesized that some of these traits would indicate unobserved conditions in a unidirectional way. That is to say, the latent variable “insect body condition” was composed of the indicator variables insect weight and development time^105^.

The response variable (outcome at the apex of the model) was an indicator of insect fitness. For males, we used relative fat body content and relative investment in muscles; and for females, we use initial egg load (relative to body volume) and fertility as proxies of reproductive performance. We additionally employed modification indices to check for pathways not included in initial models. The standardized total effects included in the supplementary material (Tables S2.b and S3.b) were calculated with the software AMOS^106^. The graphical output for SEM was produced using the library semPlot^107^. Further graphs for visualization of the main effects on specific response variables (fertility, initial egg load, fat body volume) were created using the ggplot2 library^108^.

### Ethic statement

All experiments conducted complied with current German laws.

## Supplementary information

Supplementary Information.

Supplementary Information2.

## Data Availability

All data generated and analysed during this study are published in Figshare (10.6084/m9.figshare.11347436).
