# Supplementary material for: Direct and indirect effects of plant diversity and phenoxy herbicide application on the development and reproduction of a polyphagous herbivore
Source: Sci Rep. 2020 Apr 29;10:7300. doi: 10.1038/s41598-020-64252-5 (PMC7190834; doi:10.1038/s41598-020-64252-5)
Supplement: Supplementary file 1 — Supplementary Information. [file 41598_2020_64252_MOESM1_ESM.pdf]

Direct and indirect effects of plant diversity and phenoxy herbicide application on the development and reproduction of a polyphagous herbivore

Yeisson Gutiérrez, David Ott, Christoph Scherber

Fig S1. Experimental design and summary of measured response variables

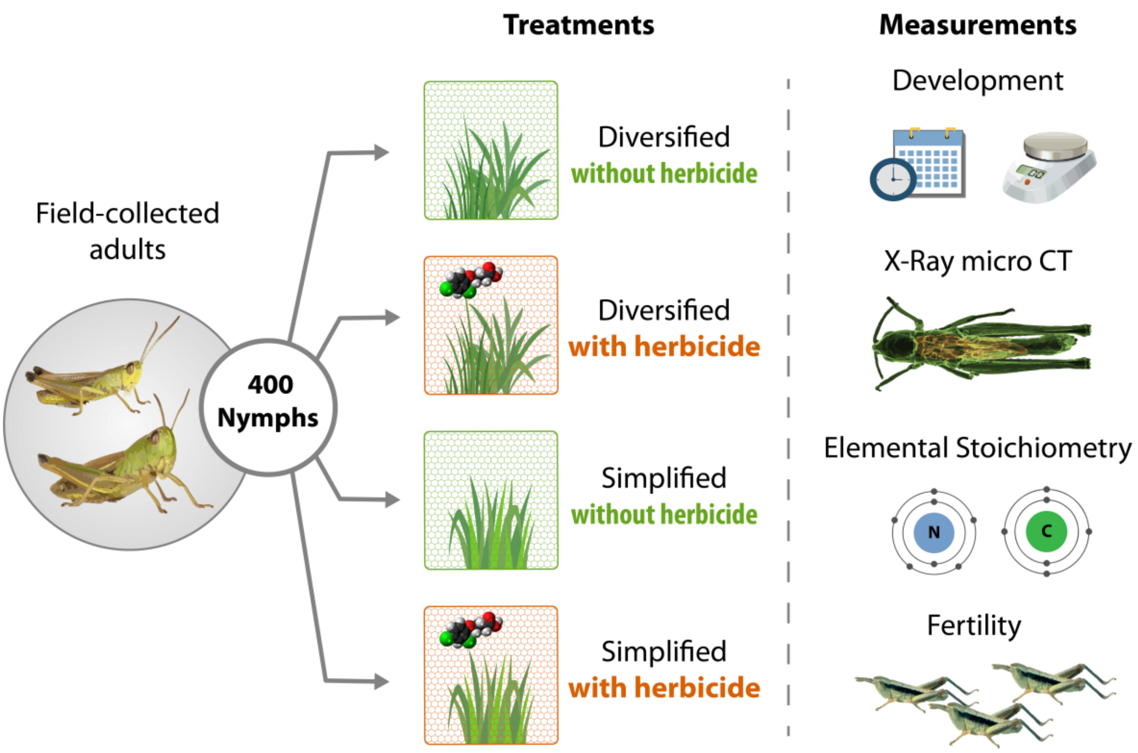

Fig S2. Layout of the position of the experimental cages in the field

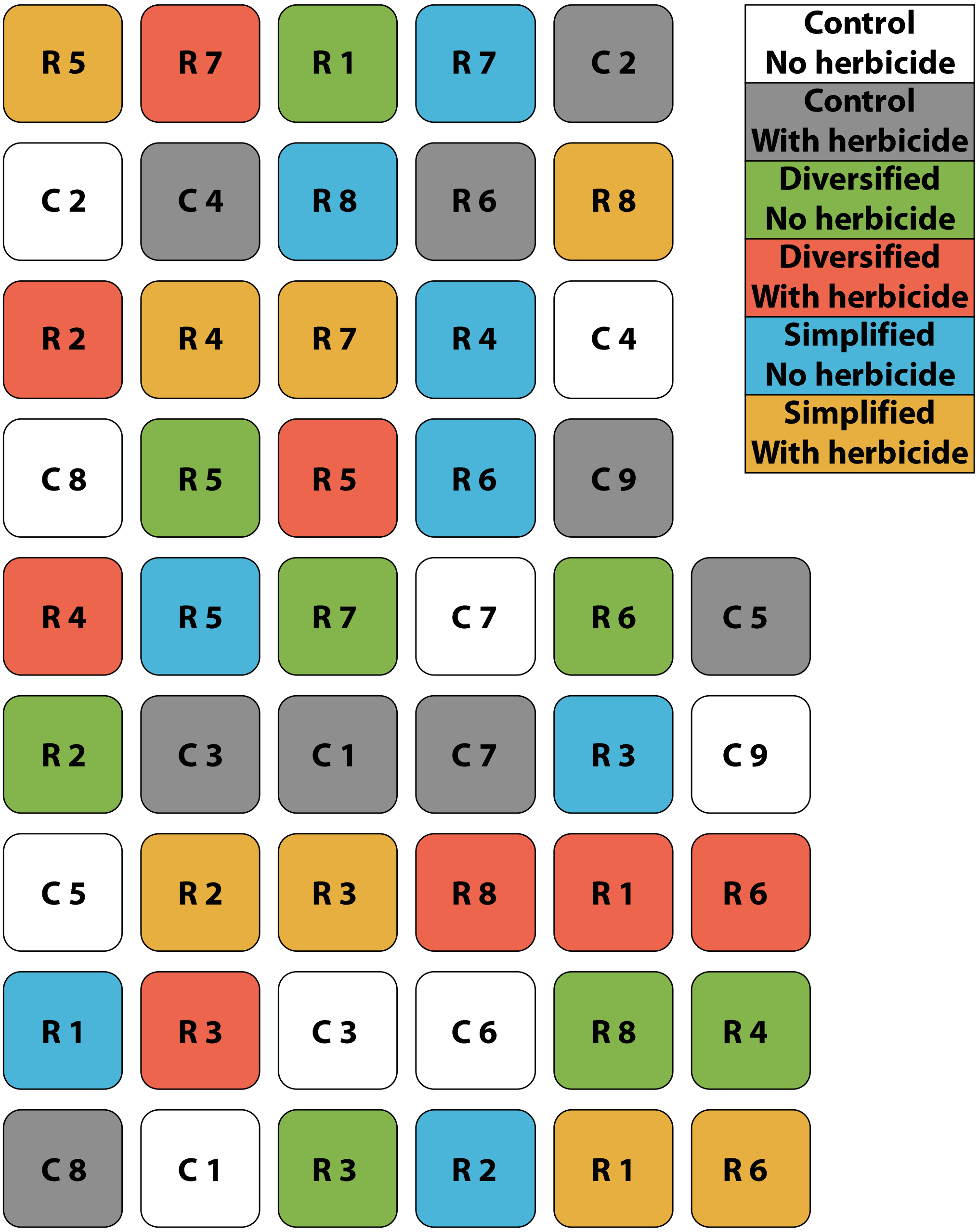

Fig. S3. Plant biomass was affected by herbicide application in a sex-specific manner. *Dactylis glometara* (Poaceae) and *Trifolium repens* (Fabaceae) increased their biomass when treated with a phenoxy herbicide

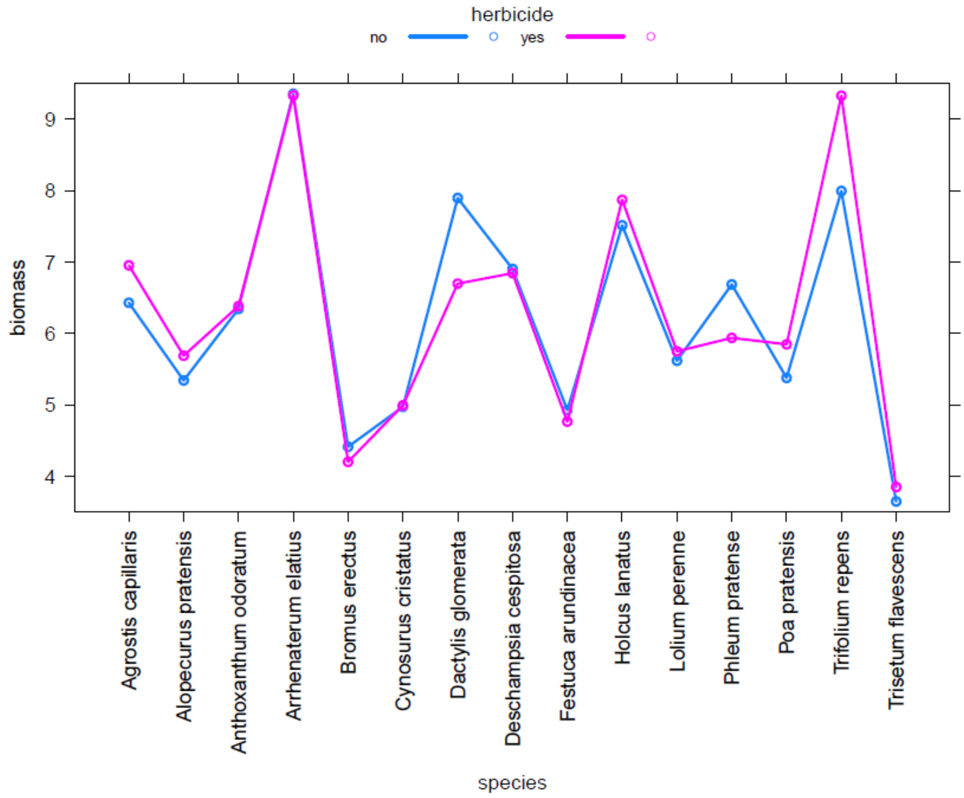

Fig. S4. Male *P. parallelus* relative investment into muscle tissue was indirectly affected by plant community diversity (see Table S3)

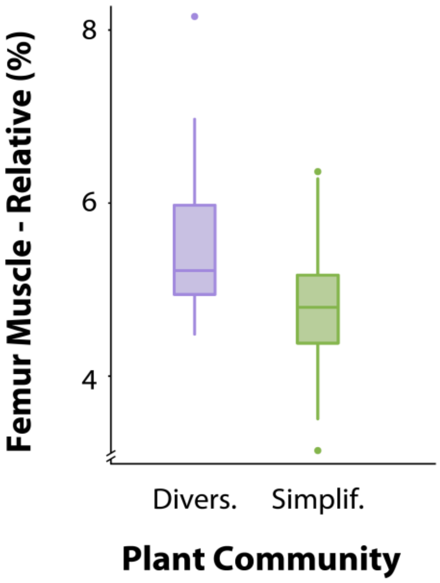

Table S1. Pool of plant species used for the experiment and experimental communities (assembled at random)

**Simplified 1**	
Grass 1	*Alopecurus pratensis*
Grass 2	*Poa pratensis*
Legume	*Trifolium repens*

**Simplified 5**	
Grass 1	*Anthoxanthum odoratum*
Grass 2	*Dactylis glomerata*
Legume	*Trifolium repens*

**Simplified 2**	
Grass 1	*Phleum pratense*
Grass 2	*Holcus lanatus*
Legume	*Trifolium repens*

**Simplified 6**	
Grass 1	*Arrhenaterum elatius*
Grass 2	*Trisetum flavescens*
Legume	*Trifolium repens*

**Simplified 3**	
Grass 1	*Deschampsia cespitosa*
Grass 2	*Bromus erectus*
Legume	*Trifolium repens*

**Simplified 7**	
Grass 1	*Agrostis capillaris*
Grass 2	*Cynosurus cristatus*
Legume	*Trifolium repens*

**Simplified 4**	
Grass 1	*Lolium perenne*
Grass 2	*Festuca arundinacea*
Legume	*Trifolium repens*

**Simplified 8**	
Grass 1	*Arrhenaterum elatius*
Grass 2	*Poa pratensis*
Legume	*Trifolium repens*

**Diversified 1**	
Grass 1	*Trisetum flavescens*
Grass 2	*Agrostis capillaris*
Grass 3	*Cynosurus cristatus*
Grass 4	*Dactylis glomerata*
Grass 5	*Deschampsia cespitosa*
Grass 6	*Festuca arundinacea*
Grass 7	*Holcus lanatus*
Grass 8	*Lolium perenne*
Legume	*Trifolium repens*

**Diversified 5**	
Grass 1	*Alopecurus pratensis*
Grass 2	*Anthoxanthum odoratum*
Grass 3	*Arrhenaterum elatius*
Grass 4	*Bromus erectus*
Grass 5	*Dactylis glomerata*
Grass 6	*Deschampsia cespitosa*
Grass 7	*Holcus lanatus*
Grass 8	*Lolium perenne*
Legume	*Trifolium repens*

Diversified 2	
Grass 1	*Alopecurus pratensis*
Grass 2	*Anthoxanthum odoratum*
Grass 3	*Arrhenaterum elatius*
Grass 4	*Cynosurus cristatus*
Grass 5	*Dactylis glomerata*
Grass 6	*Holcus lanatus*
Grass 7	*Poa pratensis*
Grass 8	*Trisetum flavescens*
Legume	*Trifolium repens*

Diversified 3	
Grass 1	*Alopecurus pratensis*
Grass 2	*Agrostis capillaris*
Grass 3	*Arrhenaterum elatius*
Grass 4	*Cynosurus cristatus*
Grass 5	*Holcus lanatus*
Grass 6	*Lolium perenne*
Grass 7	*Phleum pratense*
Grass 8	*Poa pratensis*
Legume	*Trifolium repens*

Diversified 4	
Grass 1	*Alopecurus pratensis*
Grass 2	*Agrostis capillaris*
Grass 3	*Bromus erectus*
Grass 4	*Cynosurus cristatus*
Grass 5	*Deschampsia cespitosa*
Grass 6	*Festuca arundinacea*
Grass 7	*Phleum pratense*
Grass 8	*Trisetum flavescens*
Legume	*Trifolium repens*

Diversified 6	
Grass 1	*Agrostis capillaris*
Grass 2	*Anthoxanthum odoratum*
Grass 3	*Arrhenaterum elatius*
Grass 4	*Cynosurus cristatus*
Grass 5	*Dactylis glomerata*
Grass 6	*Deschampsia cespitosa*
Grass 7	*Phleum pratense*
Grass 8	*Trisetum flavescens*
Legume	*Trifolium repens*

Diversified 7	
Grass 1	*Anthoxanthum odoratum*
Grass 2	*Bromus erectus*
Grass 3	*Dactylis glomerata*
Grass 4	*Festuca arundinacea*
Grass 5	*Lolium perenne*
Grass 6	*Phleum pratense*
Grass 7	*Poa pratensis*
Grass 8	*Trisetum flavescens*
Legume	*Trifolium repens*

Diversified 8	
Grass 1	*Alopecurus pratensis*
Grass 2	*Arrhenaterum elatius*
Grass 3	*Bromus erectus*
Grass 4	*Deschampsia cespitosa*
Grass 5	*Festuca arundinacea*
Grass 6	*Holcus lanatus*
Grass 7	*Lolium perenne*
Grass 8	*Poa pratensis*
Legume	*Trifolium repens*

Table S2. a. Summary for structural equation model for direct and indirect effects of the experimental treatments on the performance of female *Pseudochorthippus parallelus*. P-values <0.05 are reported in bold numbers and p <0.10 in italics. SE = Standard errors. **b.** Standardized total effects for female *Pseudochorthippus parallelus*

Left-hand-side	Operator	Right-hand-side	Estimate	SE	z value	P value
**Regressions**						
Plant nitrogen	~	Herbicide	0.016	0.177	0.091	0.927
Plant nitrogen	~	Diversity	0.075	0.18	0.415	0.678
Herbivory	~	Plant nitrogen	0.337	0.193	1.745	0.081
Herbivory	~	Herbicide	-0.279	0.132	-2.108	0.035
Herbivory	~	Diversity	0.35	0.139	2.518	0.012
Insect nitrogen	~	Diversity	0.399	0.131	3.04	0.002
Lifespan	~	Diversity	-0.23	0.159	-1.448	0.148
Lifespan	~	Plant nitrogen	0.308	0.139	2.213	0.027
Body mass	~	Diversity	0.375	0.155	2.419	0.016
Body mass	~	Plant nitrogen	0.361	0.145	2.492	0.013
Body mass	~	Insect nitrogen	-0.69	0.124	-5.572	0
Body mass	~	Herbivory	-0.285	0.181	-1.578	0.115
Body volume	~	Insect nitrogen	-0.324	0.141	-2.292	0.022
Fertility	~	Lifespan	0.504	0.103	4.889	0
Fertility	~	Body mass	0.346	0.104	3.327	0.001
Fertility	~	Plant nitrogen	0.243	0.095	2.55	0.011
Fertility	~	Herbicide	0.34	0.083	4.094	0
Fertility	~	Body volume	-0.207	0.114	-1.82	0.069
Fertility	~	Egg load	-0.252	0.064	-3.96	0
Egg load	~	Body mass	0.719	0.144	5.001	0
Egg load	~	Body volume	-0.875	0.617	-1.42	0.156
**Covariance**						
Body volume	~~	Egg load	0.648	0.339	1.91	0.056
**Variances**						
Plant nitrogen			0.994	0.028	36.038	0
Herbivory			0.671	0.162	4.152	0
Insect nitrogen			0.841	0.104	8.051	0
Lifespan			0.863	0.102	8.447	0
Body mass			0.467	0.124	3.778	0
Body volume			0.895	0.092	9.754	0
Fertility			0.361	0.067	5.359	0
Egg load			1.05	0.814	1.29	0.197

	Diversity	Herbicide	Plant nitrogen	Insect nitrogen	Herbivory	Body volume	Body mass	Egg load	Lifespan
**Plant nitrogen**	0.075	0.016	0	0	0	0	0	0	0
**Insect nitrogen**	0.399	0	0	0	0	0	0	0	0
**Herbivory**	0.375	-0.274	0.337	0	0	0	0	0	0
**Insect lifespan**	-0.207	0.005	0.308	0	0	0	0	0	0
**Body volume**	-0.129	0	0	-0.324	0	0	0	0	0
**Body mass**	0.02	0.084	0.265	-0.69	-0.285	0	0	0	0
**Egg load**	0.127	0.06	0.19	-0.213	-0.205	-0.875	0.719	0	0
**Fertility**	-0.085	0.361	0.442	-0.118	-0.047	0.013	0.165	-0.252	0.504

Table S3. a. Summary for structural equation model for direct and indirect effects of the experimental treatments on the performance of male *Pseudochorthippus parallelus*. P-values <0.05 are reported in bold numbers and p <0.10 in italics. SE = Standard errors. **b.** Standardized total effects for male *Pseudochorthippus parallelus*

**Left-hand-side**	**Operator**	**Right-hand-side**	**Estimate**	**SE**	**z value**	**P value**
**Regressions**						
Plant nitrogen	~	Herbicide	0.016	0.177	0.091	0.927
Plant nitrogen	~	Diversity	0.075	0.18	0.415	0.678
Herbivory	~	Plant nitrogen	0.337	0.193	1.745	0.081
Herbivory	~	Herbicide	-0.279	0.132	-2.108	0.035
Herbivory	~	Diversity	0.35	0.139	2.518	0.012
Insect nitrogen	~	Plant nitrogen	0.158	0.135	1.169	0.242
Insect nitrogen	~	Herbivory	-0.155	0.129	-1.194	0.233
Lifespan	~	Diversity	0.089	0.174	0.513	0.608
Lifespan	~	Plant nitrogen	0.355	0.217	1.637	0.102
Lifespan	~	Insect nitrogen	-0.156	0.128	-1.213	0.225
Body mass	~	Diversity	-0.281	0.142	-1.982	0.047
Body mass	~	Plant nitrogen	0.202	0.16	1.261	0.207
Body volume	~	Diversity	-0.423	0.143	-2.952	0.003
Body volume	~	Plant nitrogen	0.299	0.108	2.774	0.006
Muscle investment	~	Lifespan	-0.195	0.131	-1.487	0.137
Muscle investment	~	Body mass	-0.204	0.146	-1.401	0.161
Muscle investment	~	Fat body	0.593	0.088	6.712	>0.001
Fat body	~	Body mass	0.214	0.144	1.489	0.136
Fat body	~	Insect nitrogen	-0.249	0.146	-1.708	0.088
Fat body	~	Body volume	-0.551	0.095	-5.772	>0.001
Fat body	~	Diversity	0.226	0.132	1.715	0.086
**Variances**						
Plant nitrogen			0.994	0.028	36.039	0
Herbivory			0.671	0.162	4.152	0
Insect nitrogen			0.969	0.042	23.259	0
Lifespan			0.847	0.15	5.65	0
Body mass			0.889	0.1	8.881	0
Body volume			0.751	0.136	5.502	0
Muscle investment			0.587	0.094	6.262	0
Fat body			0.488	0.104	4.706	0

	Diversity	Herbicide	Plant nitrogen	Herbivory	Insect nitrogen	Body mass	Body volume	Lifespan	Fat body
**Plant nitrogen**	0.075	0.016	0	0	0	0	0	0	0
**Herbivory**	0.375	-0.274	0.337	0	0	0	0	0	0
**Insect nitrogen**	-0.046	0.045	0.106	-0.155	0	0	0	0	0
**Body mass**	-0.266	0.003	0.202	0	0	0	0	0	0
**Body volume**	-0.4	0.005	0.299	0	0	0	0	0	0
**Lifespan**	0.123	-0.001	0.339	0.024	-0.156	0	0	0	0
**Fat body**	0.401	-0.013	-0.148	0.038	-0.249	0.214	-0.551	0	0
**Muscle investment**	0.268	-0.008	-0.195	0.018	-0.117	-0.077	-0.327	-0.195	0.593
