# Supplementary material for: Direct and indirect effects of plant diversity and phenoxy herbicide application on the development and reproduction of a polyphagous herbivore
Source: Sci Rep. 2020 Apr 29;10:7300. doi: 10.1038/s41598-020-64252-5 (PMC7190834; doi:10.1038/s41598-020-64252-5)
Supplement: Supplementary file 2 — Supplementary Information2. [file 41598_2020_64252_MOESM2_ESM.docx]

**Supplementary information**

**Fig S1.** Experimental design and summary of measured response variables

**Fig S2.** Layout of the position of the experimental cages in the field

**Fig. S3.** Plant biomass was affected by herbicide application in a sex-specific manner. *Dactylis glometara* (Poaceae) and *Trifolium repens* (Fabaceae) increased their biomass when treated with a phenoxy herbicide

**Fig. S4.** Male *P. parallelus* relative investment into muscle tissue was indirectly affected by plant community diversity (see Table S3)

**Table S1.** Pool of plant species used for the experiment and experimental communities (assembled at random)

**Table S2. a.** Summary for structural equation model for direct and indirect effects of the experimental treatments on the performance of female *Pseudochorthippus parallelus*. P-values <0.05 are reported in bold numbers and p <0.10 in italics. SE = Standard errors. **b.** Standardized total effects for female *Pseudochorthippus parallelus*

**Table S3.** **a.** Summary for structural equation model for direct and indirect effects of the experimental treatments on the performance of male *Pseudochorthippus parallelus*. P-values <0.05 are reported in bold numbers and p <0.10 in italics. SE = Standard errors. **b.** Standardized total effects for male *Pseudochorthippus parallelus*
